# Molecular networking-based lipid profiling and multi-omics approaches reveal new contributions of functional vanilloids to gut microbiota and lipometabolism changes

**DOI:** 10.1016/j.fochms.2022.100123

**Published:** 2022-07-18

**Authors:** Hiroyuki Hattori, Akihiro Moriyama, Tomoki Ohno, Takahiro Shibata, Hitoshi Iwahashi, Tohru Mitsunaga

**Affiliations:** a Asian Satellite Campuses Institute, Nagoya University, Nagoya 464-8601, Japan; b Graduate School of Bioagricultural Sciences, Nagoya University, Nagoya 464-8601, Japan; c Research Institute of Science for Safety and Sustainability, National Institute of Advanced Industrial Science and Technology, Tsukuba 305-8569, Japan; d Faculty of Applied Biological Sciences, Gifu University, Gifu 501-1193, Japan

**Keywords:** Grains of paradise, Vanilloid, Anti-obesity, Gut microbiota, 16S rRNA gene amplicon sequencing, Lipid molecular networking

## Abstract

Obesity is now recognized as an epidemic, requiring rapid intervention. We previously demonstrated that vanilloids from the African spice known as Grains of Paradise (GOP) exhibit strong anti-obesity effects. Here, the molecular mechanism behind the obesity prevention property of the GOP extract was investigated by employing molecular networking-based lipid profiling and 16S rRNA sequencing. Administration of either GOP extract or two of its components, 6-paradol and 6-gingerol, reversed the unbalanced gut microbiota composition induced by a high-fat diet (HFD) with a decrease in Firmicutes / Bacteroidetes ratio and increase in genera *Bifidobacterium* and *Akkermansia*. Moreover, GOP extract ameliorated abnormal fatty acid metabolism in gut bacteria. Furthermore, lipid molecular networking revealed differences in lipid composition, such as triglycerides and bile acids, in the feces. These results suggest that 6-paradol and 6-gingerol can restore an unhealthy gut environment and prevent lipid absorption, a possible consequence of the reconstruction of the gut microbiota.

## Highlights

• Vanilloids from Grains of Paradise (GOP) exhibited anti-obesity effects.
• Molecular mechanism investigated using multi-omics approaches.
• GOP extract and its vanilloids restored unbalanced gut microbiota in HFD mice.
• GOP extract and its vanilloids improved F/B ratio and increased *Bifidobacterium* levels.
• GOP extract improved fecal lipid content.

## 1 Introduction

Obesity is a complex health problem caused by abnormal fat accumulation that can be associated with numerous comorbidities (Chooi et al., 2019). Many studies have shown that obesity is largely linked to low-grade chronic inflammation and intestinal dysbiosis, which occur as serious symptoms of insulin resistance, fatty liver disease, cardiovascular disease, and cancer (Chang et al., 2015). A high prevalence of obesity is currently considered a major risk factor for health problems. Therefore, it is imperative to clarify the pathophysiology of obesity in contemporary society.

Several recent studies have shown that edible plants have critical potential to prevent obesity. For instance, spices such as chili pepper, rosemary, and cinnamon have been reported to exert anti-obesity effects by reducing subcutaneous and visceral fat and promoting lipid metabolism in various tissues (Cao et al., 2019, Lu et al., 2018). We have also reported that the dried seeds of *Aframomum melegueta*, a valuable spice also known as Grains of Paradise (GOP), and its constituents, 6-paradol and 6-gingerol, showed anti-obesity properties and lower hepatic lipid concentrations in high-fat diet (HFD)-fed mice (Hattori et al., 2021).

As research on gut microbiota progresses with the development of analytical technology, the relationship between obesity and gut flora has been uncovered (Turnbaugh et al., 2009, Turnbaugh et al., 2006) beginning with the study by Gordon in 2005. Gut microbiota in humans and mice is mostly composed of Firmicutes, Bacteroidetes, Actinobacteria, and Proteobacteria. In obese humans and rodents, the proportion of Firmicutes is increased, whereas that of Bacteroidetes is decreased (The Human Microbiome Project Consortium, 2012). The personal gut flora is established in childhood and then reaches a more defined composition upon influences from genetic and/or environmental factors, including dietary habits. Evidence suggests that an altered gut microbiota composition may be involved in the development of obesity (Ursell et al., 2012).

In spite of their reported beneficial effects, the molecular mechanism behind the anti-obesity property of the GOP vanilloids remains unclear. We hypothesized that GOP extract and its vanilloids administration can determine changes in the gut microbiota composition, thus affecting lipid absorption and metabolism. Therefore, here, we examined the effect of GOP extract, 6-paradol, 6-gingerol, and 6-shogaol on gut microbiota and lipid metabolism using multi-omics approaches, lipidomics, and 16S rRNA gene amplicon sequencing.

## 2 Materials and methods

### 2.1 Animal experiments

Animal experiments were performed according to our previous procedure (Hattori et al., 2021). Briefly, five-week-old male ddY mice were purchased from Japan SLC Inc., Hamamatsu, Japan, and acclimated for a week in a conventional environment (25 ± 1 °C and 12/12 h light–dark cycle). Mice were then divided into two control groups (normal diet: ND; high-fat diet: HFD) (Table S1) and four test groups (HFD + GOP extract (50 mg/kg body weight), HFD + 6-paradol, HFD + 6-gingerol, and HFD + 6-shogaol (30 mg/kg body weight for each)) (n = 6–8), which were orally administered the test samples once daily for two weeks. Three animals were placed in each cage. The fecal samples from each mouse were collected and immersed in Tris-EDTA (TE) buffer (10 mM Tris-HCl, 1 mM EDTA, pH 8.0) (NACALAI TESQUE Inc., Kyoto, Japan) to prevent DNA degradation and stored at −30 °C until use for 16S rRNA gene amplicon sequencing and lipidomics. At the end of the experiment, mice were deprived of food and water for 8 h, sacrificed by exsanguination after being anesthetized with isoflurane, and their livers were collected for lipid analysis. All animal experiments were approved and overseen by the Gifu University Animal Care and Use Committee (Approval No. 2019–218).

### 2.2 Lipid analysis of hepatic tissue

Total hepatic lipids were extracted using the Bligh and Dyer method. Liver tissue (40 mg) was homogenized with 0.1 M acetic acid, methanol, and chloroform (4:10:5) (FUJIFILM Wako Pure Chemical Corporation, Osaka, Japan) at 4,000 rpm (2 × 1 min) and left at room temperature for 10 min. The homogenate was added to 300 μL of chloroform and homogenized at 4,000 rpm for 1 min, followed by centrifugation at 2300 × g for 10 min. The organic layers were used to analyze lipid parameters. To determine the concentrations of hepatic total cholesterol (TC) and triglycerides (TG), colorimetric enzymatic assays were conducted by using TC and TG E-test kits, respectively (FUJIFILM Wako Pure Chemical Corporation, Osaka, Japan).

### 2.3 16S rRNA gene amplicon sequencing and gut microbiota analysis

DNA was extracted from the collected feces using the Extrap Soil DNA Kit Plus ver. 2 (NIPPON STEEL & SUMIKIN Eco-Tech Corporation, Tokyo, Japan). The bacterial gene was amplified using the primers 341F/805R targeting the 16S rRNA V3–V4 region (Klindworth et al., 2013) and 2X KAPA HiFi HotStart ReadyMix (Kapa Biosystems, MA, USA). An index sequence of six bases was added to the forward primer, and seven index sequences were used, allowing the pooling of multiple samples together. PCR products were sequenced using Illumina MiSeq (Illumina Inc., Tokyo, Japan). Sequence analysis was performed according to the method described by Nishioka et al. (2021). The output Fastq file was demultiplexed into each sample based on the index sequence. After separation, lead quality filtering was performed using a fastp (Chen et al., 2018). Sequence analysis was performed based on filtered reads using QIIME2 2021.4 (Bolyen et al., 2019). Furthermore, DADA2 (Callahan et al., 2016) was used for sequence denoising, and amplicon sequence variants (ASVs) were created. Clustering analysis was conducted based on the unweighted pair group method with arithmetic mean (UPGMA) and using the Bray-Curtis dissimilarity. The clusters were visualized using the “hclust” package in R (version 4.0.2) (Murtagh and Legendre, 2014). PICRUSt2 (Douglas et al., 2019) was used to predict the gene function of the bacterial flora based on the representative sequence and number of reads output using QIIME2. Metabolic pathways were referenced to MetaCys database.

### 2.4 The quantitative PCR (qPCR) analysis

The extracted fecal-derived DNA was amplified using the THUNDERBIRD™ SYBR® qPCR Mix (Toyobo Co., ltd., Osaka, Japan) on a StepOne Plus™ system (Applied Biosystems, CA, USA). The PCR conditions were the following: 95 °C for 1 min, followed by 45 cycles at 95 °C for 15 s, 53 °C for 30 s, and 72 °C for 60 s. To calculate the relative abundance of each bacterium, the data were analyzed using the ΔΔCt method, with universal bacterial primers as the reference marker for total bacteria. The qPCR primer sequences are listed in Table S2.

### 2.5 Lipidomics

Lipid extraction was conducted as previously reported with slight modifications (Yasuda et al., 2020). Two pieces of zirconia beads and 100 μL of methanol were added to the fecal sample (10 mg) and homogenized at 4000 rpm for 20 s (Tomy Seiko Co. ltd., Tokyo, Japan). The homogenate was left at room temperature for 1 h, after which 100 μL of chloroform was added to 200 μL of the fecal suspension. The mixture was homogenized twice under identical conditions and incubated at room temperature for 1 h. Twenty microliters of MilliQ water were added to the suspension, which was then homogenized, incubated for 10 min, and centrifuged at 14500 rpm for 5 min. The supernatant was collected and 10 μL was dried under vacuum. The fecal lipid extract was resolved in 70 μL of MeOH and analyzed using a liquid chromatography (LC) system coupled with a quadrupole time-of-flight mass spectrometry (Q-TOF-MS, Agilent 6520 Accurate-Mass Q-TOF LC/MS System with Agilent 1100 Series HPLC, Agilent Technologies Inc., Tokyo, Japan) for untargeted lipidomics.

Lipids included in the feces were separated on a Shim-pack Scepter C18-120 column (3.0 × 100 mm, 1.9 μm) (Shimadzu GLC ltd., Tokyo, Japan) with a gradient elution consisting of mobile phase A (60:40 H_2_O: acetonitrile (ACN) in 10 mM ammonium formate and 0.1 % formic acid) and B (90:10 isopropanol (IPA) /ACN also with 10 mM ammonium formate and 0.1 % formic acid). The LC gradient was as follows: initiation at 20 % B (0 to 2 min); increase to 40 % B (from 2 to 8 min); increase to 50 % B (from 8 to 11 min); increase to 55 % B (from 11 to 17 min); increase to 65 % B (from 17 to 23 min); increase to 70 % B (from 23 to 28 min); increase to 75 % B (from 28 to 33 min); increase to 97 % B (from 33 to 39 min); maintained 97 % B (from 39 to 47 min); decreased to 20 % B, and maintained for 10 min before the next injection; column temperature was 45 °C; the flow rate was set at 0.3 mL/min. Ammonium formate and formic acid were purchased from FUJIFILM Wako Pure Chemical Corporation, Osaka, Japan, and ACN and IPA were purchased from KANTO KAGAKU, Tokyo, Japan.

The MS profiling details were as follows: data-dependent MS/MS acquisition (DDA) mode at high resolution was used. The parameters were MS1 and MS2 mass ranges, *m*/*z* 100–1700 and 50–1700, respectively; MS1 and MS2 acquisition times, 1000 and 333 ms, respectively; collision energy (use slope), 4 V/100 Da and offset 15 V; cycle time, 2.1 s; gas temperature was 330 °C; VCap was 3300 V; and Fragmentor was set at 175 V.

### 2.6 Feature-based molecular networking

Untargeted LC-MS/MS data were analyzed using MS-DIAL ver. 4.70 to annotate the lipid subclasses with a slight modification of reported parameters (Naoe et al., 2019): (Data collection) MS1 and MS2 tolerances: 0.01 and 0.025 Da respectively; RT begin: 0 min; RT end: 100 min; mass range start: 0 Da; mass range end: 2000 Da; maximum charged number: 2; (Peak detection) minimum peak height: 500; mass slice width: 0.1 Da; smoothing method: linear weighted moving average; smoothing level: 3 scan; minimum peak width: 5 scan; (MS2Dec) sigma window value: 0.5; MS2 abundance cut off: 0 amplitude; exclude after precursor: true; keep isotope until: 0.5 Da; keep isotope MS2Dec: false; (Identification) Lipid database setting: check all; retention time tolerance: 100 min; accurate mass tolerances (MS1) and (MS2): 0.01 and 0.05 Da; identification score cut off: 80 %; using retention time for scoring and filtering: false; relative abundance cut off: 0 %; only report the top hit: false; (Alignment) retention time tolerance: 0.1 min; MS1 tolerance: 0.015 Da; peak count filter: 0 %. The other parameters were used as default. The results were visualized using the Cytoscape 3.90 ver. Furthermore, principal component analysis (PCA) was performed using the program “procomp” in R version 4.0.2.

### 2.7 Statistical analysis

Statistical analyses of group comparisons were performed using GraphPad Prism 9, and the comparisons were analyzed using one-way ANOVA followed by Tukey’s multiple comparison test.

## 3 Results and discussion

### 3.1 GOP extract and its components improve impaired hepatic lipid metabolism

**Fig. 1:**
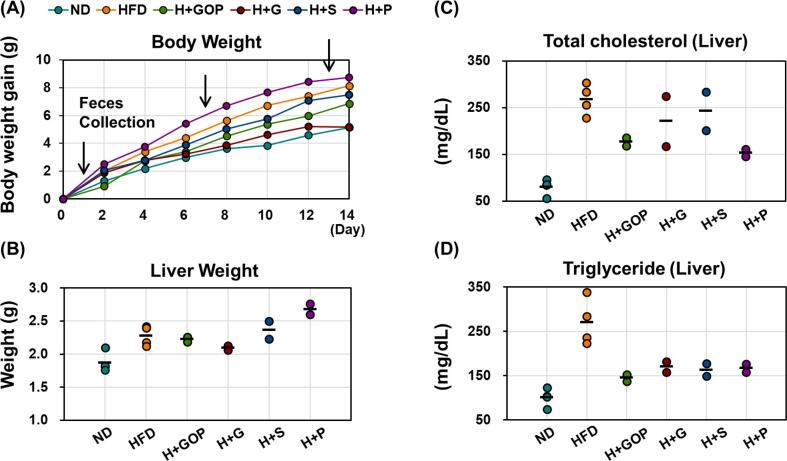
**GOP extract and its components ameliorate obesity-related parameters.** Mice were fed either a normal diet (ND, n = 4), high-fat diet (HFD or H, n = 4), HFD + 50 mg/kg body weight of GOP extract (n = 2), or HFD + 30 mg/kg body weight of either 6-paradol (P, n = 2), 6-gingerol (G, n = 2), or 6-shogaol (S, n = 2) for two weeks. (**A**) Bodyweight curve, (**B**) Liver weight, (**C**) Hepatic total cholesterol, and (**D**) hepatic triglyceride concentrations were measured.

According to our previous report (Hattori et al., 2021), several physiological parameters, including body and liver weights, total cholesterol and triglyceride values, and food intake, were analyzed to determine the effect of GOP extract and its components (Fig. 1 and Table S3). In our study, high-fat diet (HFD)-fed mice were orally administered either GOP extract, 6-paradol, 6-gingerol, or 6-shogaol for 2 weeks. HFD mice presented values for each parameter indicative of obese conditions, whereas normal diet (ND) mice showed healthy conditions. Hepatic TC and TG levels were decreased in the GOP extract and 6-paradol groups. Our findings indicate that treatment with GOP extract and its vanilloids can reduce body weight and TC and TG levels in the liver, thus ameliorating HFD-induced obesity in mice.

### 3.2 GOP extract and its components altered gut bacterial diversity

**Fig. 2:**
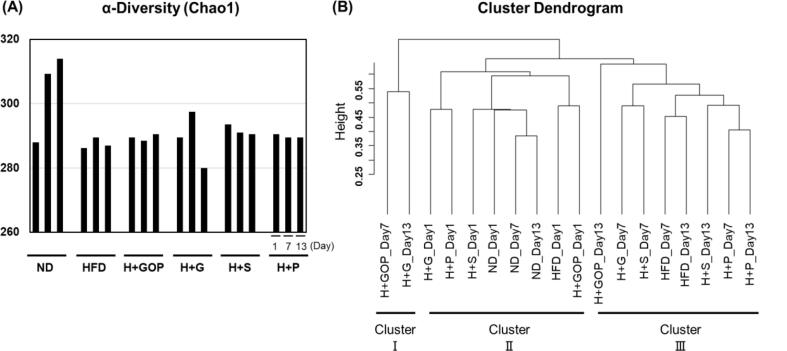
**GOP extract and its components altered gut microbiota composition in HFD-fed mice.** (**A**) Chao1 index in the normal diet (ND, n = 4), high-fat diet (HFD, n = 4), HFD + GOP (n = 2), HDF + either 6-paradol (P, n = 2), 6-gingerol (G, n = 2), or 6-shogaol (S, n = 2) groups at 1, 7, 13 days, (**B**) Hierarchical clustering by the unweighted pair group method with arithmetic mean (UPGMA) based on Bray-Curtis dissimilarity between each sample’s amplicon sequence variants (ASVs) composition.

To investigate bacterial diversity in the intestinal tract, DNA was extracted from feces of the mice under the different diet types and treatment and analyzed through bacterial 16S rRNA sequencing. The α and β diversities were used to elucidate the diversity of microbial composition, whereas Chao1 index was calculated based on the number of observed bacterial species. ND mice showed an increase in the Chao1 value on day 13. Although no detectable differences were observed in the other experimental groups, a reduction in the Chao1 value on day 13 was observed after 6-gingerol treatment (Fig. 2A). According to previous studies reporting a decrease in the Chao1 index-related diversity associated with HFD intake (Campbell et al., 2019, Song et al., 2021), our results showed that, when compared with the ND group, HFD intake modified the microbial diversity and, among the tested compounds, 6-gingerol changed the abundance of certain bacteria over two weeks, suggesting a disrupted gut environment.

To determine the differences in bacterial structures, the experimental groups were then clustered based on their β diversity by employing UPGMA and using the Bray-Curtis dissimilarity (Fig. 2B). Three clusters (Ⅰ–III) were generated with the HFD + GOP extract (H + GOP) at day 7 and HFD + 6-gingerol (H + G) at day 13 (Cluster I) being the most distant from the other groups. In addition, the ND group on days 1, 7, and 13 belonged to the same cluster together with the other groups on day 1 (Cluster II), and the H + GOP group on day 13 differed in the microbial diversity from the other groups, although most of the HFD-fed groups were included in Cluster III (Fig. 2B). The clustering analysis suggested that although the intestinal microbiota composition was similar in all groups at the beginning of the experiment, it was noticeably affected by the treatment after two weeks, with the major effect being observed for the H + GOP and H + G groups.

### 3.3 GOP extract and its components impact microbiota composition at the phyla level

**Fig. 3:**
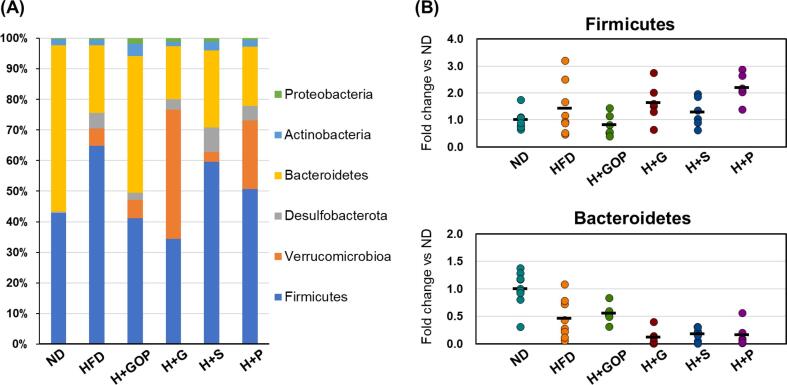
**Effects of the GOP extract and its components on gut microbiota compositions (phyla level).** (**A**) Taxonomic classification and (**B)** qPCR-based specific detection of Firmicutes and Bacteroidetes in normal diet (ND), high-fat diet (HFD), HFD + GOP, HDF + either 6-paradol (P, n = 2), 6-gingerol (G, n = 2), or 6-shogaol (S) groups. The black lines indicate the average values.

We also analyzed the bacterial composition at the phylum level by taxonomic annotation of the sequences on day 13 (Fig. 3A). The relative abundance of Firmicutes was increased, whereas a reduction was observed in the Bacteroidetes in HFD-fed mice as compared to the ND group. However, GOP extract and 6-gingerol intake decreased the abundance of Firmicutes (41.1 % and 34.4 %, respectively). The relative abundance of Bacteroidetes was increased by the GOP extract to the level observed in ND group. Moreover, Desulfobacterota and Verrucomicrobiota were found to be enhanced in all HFD-fed mice, and the abundance of Verrucomicrobiota upon 6-paradol and 6-gingerol administration was 7.43 and 3.94 times higher than that in the HFD control mice.

Bacteroidetes and Firmicutes, two major bacterial phyla in rodents and humans, have received considerable attention because of their importance to human health (Stojanov et al., 2020). Accordingly, with previous studies reporting that an obesogenic HFD results in gut microbiota changes with increased Firmicutes to Bacteroidetes ratio (F/B), we found that F/B in HFD group is 2.93 (Table S4) (Ley et al., 2005). Additionally, the GOP extract and 6-gingerol improved the F/B ratio to 0.92 and 1.98, respectively, values similar to those observed in the healthy ND mice (F/B: 0.79), suggesting an anti-obesity effect through amelioration of the gut microbial imbalance.

Next, we quantified the presence of the bacteria *via* 16S rRNA-targeted qPCR using specific primers for the detection of Firmicutes and Bacteroidetes (Fig. 3B). The relative abundance of Firmicutes in the HFD, H + G, H + S, and H + P groups was higher than that detected in the ND group (1.42, 1.64, 1.29, and 2.20, respectively). Bacteroidetes displayed a lower abundance in these groups (0.46, 0.12, 0.17, and 0.16, respectively) as compared with ND, although the GOP extract seemed to ameliorate the unhealthy gut microbiota condition induced by the HFD, thus supporting the 16S rRNA sequencing data.

### 3.4 GOP extract and its components impact microbiota composition at the genus level

**Fig. 4:**
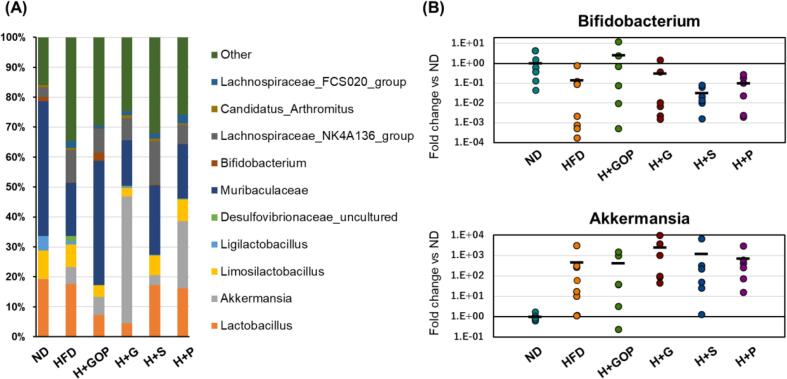
**Effects of the GOP extract and its components on gut microbiota compositions (genus level**)**.** (**A**) taxonomic composition and (**B**) qPCR-based measurement of *Bifidobacterium* and *Akkermansia* in normal diet (ND, n = 8), high-fat diet (HFD, n = 8), HFD + GOP (n = 6), HDF + either 6-paradol (P, n = 6), 6-gingerol (G, n = 6), or 6-shogaol (S, n = 6) groups. The black line represents the average values.

To clarify whether the GOP extract and its vanilloids modulate the intestinal bacterial flora, we further investigated the gut bacterial composition at the genus level using the data from the 16 s rRNA sequencing and qPCR (Fig. 4). It has been reported that *Muribaculaceae* is related to obesity (Cao et al., 2020). The relative abundance of *Muribaculaceae* in the HFD-fed mice (17.8 %) was notably higher after GOP extract supplementation (41.5 %), whereas the other treatment groups showed approximately 20 % abundance. Similarly, the ND and GOP groups presented a higher level of *Bifidobacterium* (1.5 % and 2.6 %, respectively), but not the other groups (below 0.3 %). In addition, *Desulfovibrio* was distinctive in the HFD group and *Akkemansia* was abundant in the H + G and H + P groups (42.3 % and 22.4 %, respectively) (Fig. 4A). Further quantification by qPCR was performed on *Bifidobacterium* and *Akkermansia*, the abundance of which was affected by supplementation with GOP extract and its vanilloid (Fig. 4B). Our results showed that *Bifidobacterium* was restored by GOP extract and 6-gingerol administration, which increased the abundance of this bacterial genus by 18.5- and 2.3-fold, respectively, compared with the HFD group. Zhan et al reported that the presence of *Bifidobacterium* was decreased by HFD, which is in line with our data (Zhao et al., 2021). 6-Gingerol also promoted *Akkermansia* growth, with a 5.3-fold increase compared to the HFD group. These results were consistent with the 16S rRNA sequencing data (Fig. 4A).

### 3.5 GOP extract and its components altered microbiota metabolism

**Fig. 5:**
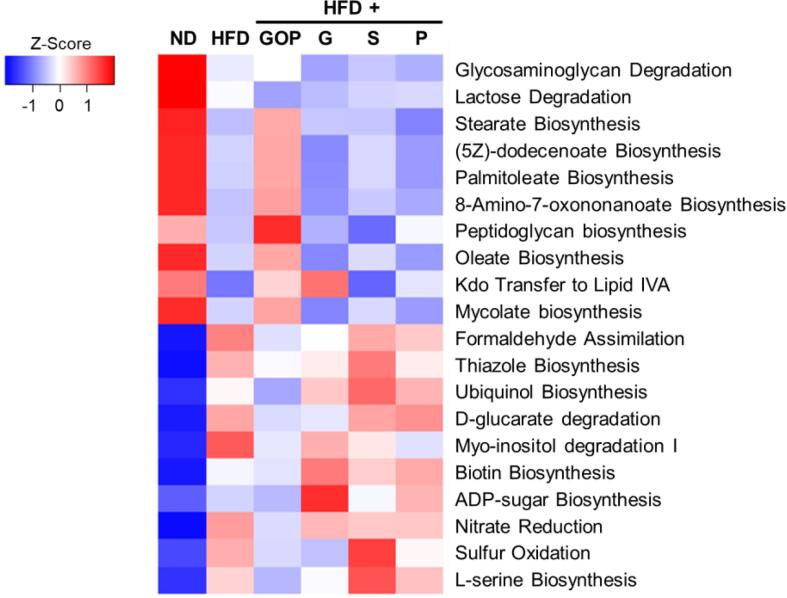
**Heatmap of the enriched and impoverished metabolic pathways of each tested group mouse.** The top ten enriched and least represented pathways were described in the heatmap. For each functional pathway, those with a relative presence of 0.01% or higher for both ND and HFD conditions were included in the analysis.

Several functional categories were estimated by gut microbiota reconstruction based on the 16S rRNA sequencing results using PICRUSt2 (Fig. 5) (Douglas et al., 2019). The top ten most and least enriched pathways from the comparison between ND and HFD mice are shown. ND and GOP group mice showed similar changes in metabolic pathways, except for the glycosaminoglycan degradation and lactose degradation pathways with the most relevant enriched MetaCyc pathways including stearate, palmitoleate, oleate, and mycolate biosynthesis. The finding suggest that the GOP extract contributes to recovery from unhealthy microbiome function caused by HFD intake. Furthermore, GOP extract and 6-gingerol supplementation increased sulfur oxidation and l-serine biosynthesis pathways, which might affect the intestinal environment.

### 3.6 GOP extract and its components altered fecal lipid contents

Based on the results of the functional pathway prediction (Fig. 5), we speculated that lipid metabolism changes occurred in response to HFD, due to the structural differences in the microbiota. A comprehensive analysis of the differences in the lipid components in the feces between the tested groups was performed.

**Fig. 6:**
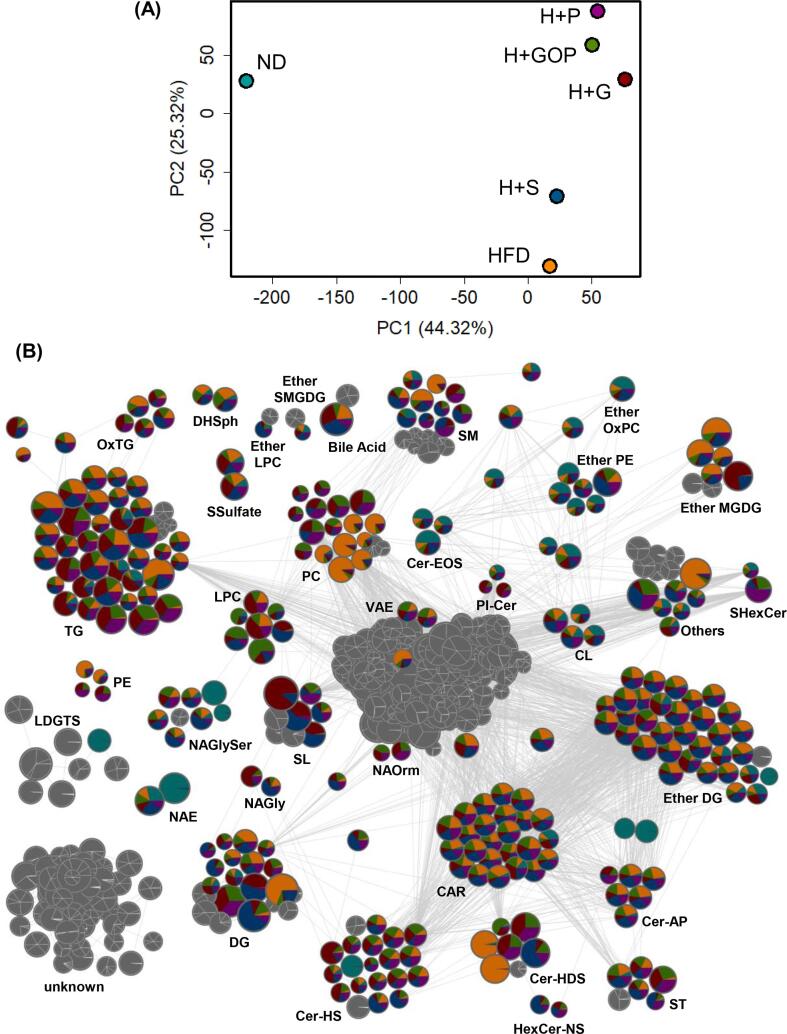
**Analysis of fecal lipid composition altered by GOP extract and its components.** (**A**) Principal component analysis (PCA) score plot based on the lipid proportion in feces. The dissimilarity of fecal lipid proportion between tested group mice is represented. (**B**) Lipid molecular networking of fecal samples from each treated mouse. Each node includes information about lipid subclasses, relative measured ion intensity, and the ratio of detected lipids in each treated mouse. The node size represents the mean number of spectra detected (n = 2, from selected samples for 16 s rRNA sequencing).

Principal component analysis (PCA) was conducted based on the identified lipids (approximately 27,000 types) and the peak area of each lipid to reveal the variation in the feces (Fig. 6A). Fecal lipid content was largely different between the ND and HFD groups. H + GOP, H + P, and H + G exhibited a similar lipid composition that was remarkably changed compared to the HFD group, which could reflect the anti-obesity effect of each tested sample. Our previous results demonstrated that 6-shogaol failed to show an anti-obesity effect after two weeks (Hattori et al., 2021). This might be explained by the fact that the lipid composition between 6-shogaol and HFD group mice was similar, although further analysis is required.

We performed untargeted lipidomics *via* LC-QTOF/MS to create a lipid molecular network using MS-DIAL and visualized it using the Cytoscape software (Fig. 6B), which detected 6067 features with MS/MS fragments, and focused on 707 signature lipid classes described in the network as nodes. To understand the metabolism of lipids affected by intestinal microbiota and/or GOP extract and its vanilloids administration, we employed a feature-based molecular networking (FBMN) approach. The nodes denote metabolic ion features and are matched if the MS/MS spectra have a high similarity, indicating the existence of the same or similar substitutes.

After lipid annotation by FBMN, triacylglycerol (TG), alkylacyl diacylglycerol (Ether DG), carnitines (CAR), diacylglycerol (DG), ceramide hydroxy fatty acid-sphingosine (Cer_HS), phosphatidylcholine (PC), sphingomyelin (SM), ceramide alpha-hydroxy fatty acid-phytosphingosine (Cer_AP), sulfonolipid (SL), *N*-acyl glycine serine (NAGlySer), alkylacyl monoglucosyl/galactosyl diacylglycerol (Ether MGDG), lysophosphatidylcholine (LPC), Hexosylceramide hydroxyfatty acid-dihydrosphingosine (Cer_HDS), alkylacyl phosphatidylethanolamine (Ether PE), and sterols (ST) were mainly included into the network.

For the TG cluster, the HFD group was the most predominant, whereas the administration of GOP extract, 6-paradol, and 6-gingerol promoted TG excretion. We also investigated the relationship between gut bacteria and lipid classes in feces and found that bile acid (ND: 46.43; HFD: 618.47; H + GOP: 321.50; H + G: 819.16; H + S: 1217.42; H + P: 628.94, calculated by the average peak area) and *Bifidobacterium* (ND: 54.5 %; HFD: 22.1 %; H + GOP: 44.8 %; H + G:17.4 %; H + S: 25.3 %; H + P: 19.3 % from sequencing data) had a negative relation. In contrast, TG (ND: 0.24; HFD: 2.71; H + GOP: 8.73; H + G:12.17; H + S: 2.32; H + P: 10.75, calculated by the average peak area) and *Akkermansia* (ND: 0.05 %; HFD: 5.69 %; H + GOP: 6.06 %; H + G: 42.33 %; H + S: 3.22 %; H + P: 22.41 % from the sequencing data) showed a positive relation.

Our data showed a decreased abundance of *Bifidobacterium* in HFD mice, reversed by the administration of GOP extract. An opposite trend was observed for bile acid excretion in the feces. It has been reported that the abundance of *Bifidobacterium* is low and fecal bile acid is high in obese humans and rodents. Additionally, intestinal bile acid is metabolized as deconjugated and dehydroxy by *Bifidobacterium* and other bacteria (Cani et al., 2008, Jia et al., 2018). Our findings suggest that the GOP extract accelerated the growth of *Bifidobacterium* and the excretion of bile acid, which might lead to the anti-obesity effect.

16S rRNA sequencing analysis showed that the relative abundance of *Akkermansia* increased with HFD feeding and further increased with 6-paradol and 6-gingerol treatments. Similarly, lipidomics results showed that fecal TG was higher in HFD mice and even higher in the H + G and H + P groups. *Akkermansia* found in healthy human feces was previously associated with obesity (Ottman et al., 2017). A proposed anti-obesity mechanism consists of *Akkermansia* contribution to TG and postprandial chylomicron clearance to avoid acute lipid overload in circulation (Xu et al., 2020). Our results also suggest that the administration of 6-paradol and 6-gingerol to mice with a disrupted gut microbiota due to HFD increases *Akkermansia*, which in turn causes fecal TG excretion, thereby exerting an anti-obesity effect. Although the main anti-obesity mechanism of GOP extract and its components appears to be the alteration of the gut microbiota, another potential mechanism might be the inhibition of lipase (Cavalcanti et al., 2022, Coronado-Cáceres et al., 2020, Shimizu et al., 2020).

## 4 Conclusion

We illustrated a possible anti-obesity mechanism of the GOP extract and its components using multi-omics approaches, molecular networking-based lipid profiling and 16S rRNA gene amplicon sequencing. GOP extract and its vanilloids, 6-paradol, and 6-gingerol obviously modulated the gut microbiota composition disrupted by HFD feeding, including *Bifidobacterium* and *Akkermansia*, which could lead to an anti-obesity effect. It was also found that the HFD + GOP extract, 6-paradol, and 6-gingerol groups had very similar fecal lipid profiles and were completely different from those of the HFD group. Lipid molecular networking combined with sequencing is a powerful method to understand microbial lipometabolism, which could be applied to discover natural resources and lead compounds with beneficial health properties.

## Declaration of Competing Interest

The authors declare that they have no known competing financial interests or personal relationships that could have appeared to influence the work reported in this paper.
