# Supplementary material for: Molecular networking-based lipid profiling and multi-omics approaches reveal new contributions of functional vanilloids to gut microbiota and lipometabolism changes
Source: Food Chem (Oxf). 2022 Jul 18;5:100123. doi: 10.1016/j.fochms.2022.100123 (PMC9309231; doi:10.1016/j.fochms.2022.100123)
Supplement: Supplementary data 1 [file mmc1.docx]

**Table S1** Dietary Composition (%). The normal diet was purchased from Oriental Yeast Co., ltd., Tokyo, Japan.

|  | ND | HFD |
| --- | --- | --- |
| Water | 7.9 | 6.3 |
| Protein | 23.1 | 18.5 |
| Fat | 5.1 | 24.1 |
| Ash | 5.8 | 4.6 |
| Fiber | 2.8 | 2.2 |
| Nitrogen-free extract | 55.3 | 44.2 |

**Table S2** Primers for amplification of the bacterial gene

| Target Genes | Primers (Forward) | Primers (Reverse) | Reference |
| --- | --- | --- | --- |
| Universal (Bacteria) | AAACTCAAAKGAATTGACGG | CTCACRRCACGAGCTGAC | **[S1]** |
| Bacteroidetes | TGAAACTYAAAGGAATTGACG | ACCATGCACCACCTGTC | **[S1]** |
| Firmicutes | CRAACAGGATTAGATACCCT | GGTAAGGTTCCTCGCGTAT | **[S1]** |
| *Akkermansia* | CTGAAGAACTCGGCACCCTT | CTTCTTCAGCTTCGGCAGGA | **[S2]** |
| *Bifidobacterium* | CGCGTCYGGTGTGAAAG | CCCCACATCCAGCATCCA | **[S2]** |

**Table S3** Total diet intake in 2 weeks of each mouse group

**Table S4** Relative abundances of Firmicutes and Bacteroidetes and their ratio in each treated group based on 16s rRNA sequencing data.


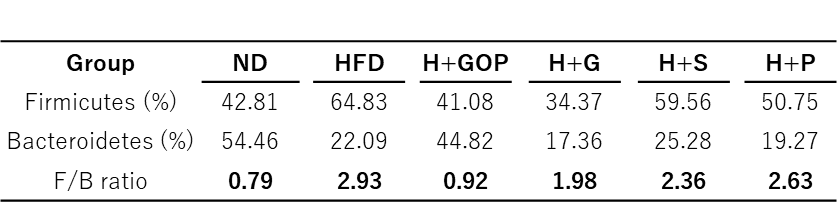


**MATERIALS AND METHODS**

***Analysis of GOP extract***

HPLC analysis (Shimadzu Corporation, Kyoto, Japan) of GOP extract and its components (6-paradol, 6-gingerol, and 6-shogaol) was performed using an Inertsil^®^ ODS-3 column (4.6 mm φ × 250 mm) with a gradient elution consisting of mobile phase A (0.05% trifluoroacetic acid (TFA) /H_2_O) and B (MeOH). The LC gradient was as follows: initiation at 50% B, increased to 100% B (from 0 to 40 min), maintained at 100% B (from 40 to 50 min), decreased to 50% B, and maintained for 10 min before the next injection; column temperature was 40 °C; flow rate was set at 1.0 mL/min; wavelength was 280 nm.

**RESULT**

***GOP extract is rich in 6-paradol, 6-gingerol, and 6-shogaol***

To detect the GOP components, the HPLC analysis was conducted (**Fig. S1**). The three major components made up more than 70% of the GOP extract, while 6-paradol and 6-gingerol each accounted for approximately 30% of the total extract content.

**Figure S1.** HPLC chromatogram (280 nm) of GOP extract, 6-paradol, 6-gingerol, and 6-shogaol.
